# Sclerostin Antibody Therapy for the Treatment of Osteoporosis: Clinical Prospects and Challenges

**DOI:** 10.1155/2016/6217286

**Published:** 2016-05-26

**Authors:** Claire MacNabb, D. Patton, J. S. Hayes

**Affiliations:** Regenerative Medicine Institute, NUI Galway, Biosciences Research Building, Corrib Village, Dangan, Galway, Ireland

## Abstract

It is estimated that over 200 million adults worldwide have osteoporosis, a disease that has increasing socioeconomic impact reflected by unsustainable costs associated with disability, fracture management, hospital stays, and treatment. Existing therapeutic treatments for osteoporosis are associated with a variety of issues relating to use, clinical predictability, and health risks. Consequently, additional novel therapeutic targets are increasingly sought. A promising therapeutic candidate is sclerostin, a Wnt pathway antagonist and, as such, a negative regulator of bone formation. Sclerostin antibody treatment has demonstrated efficacy and superiority compared to other anabolic treatments for increasing bone formation in both preclinical and clinical settings. Accordingly, it has been suggested that sclerostin antibody treatment is set to achieve market approval by 2017 and aggressively compete as the gold standard for osteoporotic treatment by 2021. In anticipation of phase III trial results which may potentially signify a significant step in achieving market approval here, we review the preclinical and clinical emergence of sclerostin antibody therapies for both osteoporosis and alternative applications. Potential clinical challenges are also explored as well as ongoing developments that may impact on the eventual clinical application of sclerostin antibodies as an effective treatment of osteoporosis.

## 1. Introduction

The identification of sclerostin as a therapeutic target and the optimisation of anti-sclerostin antibodies (Scl-mAb) have led to a vast array of preclinical studies documenting its ability to enhance bone formation, strength, and density [1, 2]. Developments have even attracted the attention of NASA with treatment potentially capable of reversing bone density deterioration experienced by astronauts during prolonged space flight [3]. The first human clinical trial continued the success story with a single injection of Scl-mAb surpassing gains in bone mineral density beyond levels expected after six months of daily teriparatide injections [4]. Amgen (romosozumab), Eli Lilly (blosozumab), and Novartis (BPS804) represent the main industrial backers of Scl-mAb therapy. In light of recent clinical trial developments, industry commentators fully expect market approval for a humanized anti-sclerostin antibody by 2017 [5]. There is little doubt that there is a serious need for effective treatment. The World Health Organization considers osteoporosis second only to cardiovascular disease as a threat to global health with over 200 million sufferers globally facing an increased risk of fracture and related complications [6]. Current approaches encompass largely anti-resorptive treatments such as bisphosphonates, selective oestrogen receptor modulators, oestrogen, and denosumab antibody therapy. Teriparatide available as either full-length (recombinant human PTH 1-84) or the active fragment (PTH 1-34) is currently the only clinically available anabolic treatment for osteoporosis. However, only PTH 1-34 is licensed for use in the USA. Moreover, there are considerable disadvantages with the use of teriparatide. For instance, the use of teriparatide and recombinant human PTH has been limited to 18–24 months in the USA and EU, respectively. Also, while initially associated with increases in bone formation eventually their use leads to a rise in markers of bone resorption. Consequently, while many existing therapies are available, new treatment options are continuously sought. In this review, we provide a perspective of the field of Scl-mAb therapy. Specifically, we examine the development of Scl-mAb through preclinical and clinical studies while assessing the strengths and potential short-comings of treatments. A commentary on areas for further research and novel future applications is also explored.

## 2. Osteoporosis and the Need for Intervention

Osteoporosis is a metabolic bone disease characterised by a significant decrease in bone mineral density (BMD) and structural changes to the bone that greatly increase the risk of fracture [7]. The condition is age-related and affects both sexes. However, it is particularly prevalent in postmenopausal women with one in three women aged over 50 likely to experience an osteoporotic fracture compared to one in five men. The increased prevalence after menopause is due to decreased oestrogen levels which accelerate bone loss. Hip fractures, in particular, have a serious impact on patient welfare. These result in reduced mobility, chronic pain, and a greatly increased level of dependence, with 10–20% of previously independent sufferers being admitted to nursing homes [8]. The social and economic cost of osteoporosis is considerable. It is estimated that 1.6 million osteoporotic hip fractures occur annually and are set to increase to 6.3 million globally by 2050 [9]. Within the EU in 2010, approximately 5.5 million men and 22 million women were estimated to have osteoporosis. Moreover, the cost of treatment for osteoporotic fractures and pharmaceutical intervention of the disease are projected at €37 billion per annum [8]. Osteoporotic fractures are further complicated by the inherent difficulties in ensuring adequate fixation of pins and screws due to diminished quality of the surrounding bone [10, 11]. The occurrence of osteoporosis is set to increase drastically with the number of sufferers in the EU alone forecasted to rise by 23% to 33.9 million by 2025 [12]. This will create a serious medical and economic challenge and highlights the need for effective strategies to deal with the issue on a global scale [13]. In order to be successful, the development of these international strategies must be built on a solid foundation of safe and effective therapies that can be applied at an individual level.

## 3. Sclerostin, Its Mode of Action and Potential as a Therapeutic Target

### 3.1. Sclerostin

The role of sclerostin in the modulation of the Wnt/*β*-catenin dependent pathway came to light specifically through the study of sclerosteosis and van Buchem disease, two rare genetic disorders associated with high levels of BMD and an associated low risk of fracture [14]. Both diseases were traced to mutations impacting a single gene, SOST, which is mainly expressed from osteocytes. Nevertheless, its expression in chondrocytes, osteoblasts, the bone marrow, heart, pancreas, liver, and some foetal tissue has been reported [15–17]. The glycoprotein sclerostin is a product of the SOST gene. The amino acid sequence of sclerostin distantly resembles that of the Cerebus/DAN (differential screening-selected gene aberrative in neuroblastoma) family of glycoproteins, defined by 190 residues with a cysteine knot-like domain. However, while DAN proteins act as classical antagonists of BMP signalling, sclerostin-BMP interactions are weak and appear to be specific to BMP-7 in osteocytes alone [18, 19]. Others suggest that sclerostin exhibits a catabolic effect on bone by increasing osteoclast production via increased osteocytic expression of receptor activator of nuclear factor kappa B (RANKL). The decline in RANKL signalling may at least in part explain the sustained decreases in bone resorption markers observed with antisclerostin therapy [20, 21].

**Figure 1:**
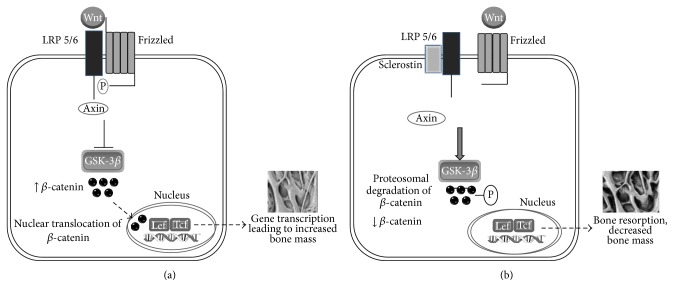
(a) Canonical Wnt signalling involves binding of Wnt to LRP5/6 and its coreceptor Frizzled resulting in the phosphorylation of LRP5/6 thus permitting Axin to bind to the receptor complex. Formation of this complex leads to inhibition of GSK3*β* which prevents degradation of *β*-catenin. Accumulation of cytosolic *β*-catenin leads to nuclear translocation where it activates target gene promoters which result in increased bone mass. (b) Sclerostin inhibition of the Wnt-canonical pathway in osteogenesis. Sclerostin binding to LRP receptor 5/6 prevents Wnt binding and formation of the Frizzled-LRP complex and thus Axin remains unphosphorylated. Downstream effects include activation of GSK3*β* resulting in phosphorylation of cytosolic *β*-catenin, thus targeting it for degradation. In the absence of *β*-catenin accumulation and subsequent nuclear translocation, osteogenesis is prevented.

Several sources of evidence implicate a prominent role for sclerostin as an antagonist of the Wnt signalling pathway [22, 23]. The Wnt gene family are largely characterised by their highly conserved glycosylated secreted proteins and can be broadly defined by canonical and noncanonical mediators. The latter typically involve cGMP-related signalling, Jun-kinase activation, and/or activation of protein kinase A. Primarily the noncanonical pathway is involved in processes such as tissue formation during development, maintenance of adult stem cells, and tumour repression [24]. Canonical Wnts are defined by their ability to stabilise *β*-catenin via Wnt ligand binding to the Frizzled (FZD) receptor and low-density lipoprotein receptor-related proteins 5 and 6 (LRP5/6). This results in the phosphorylation of LRP5/6 thus permitting Axin to bind to the receptor complex. This results in the inhibition of glycogen synthase kinase-3*β*'s (GSK-3*β*) activity, which ordinarily targets *β*-catenin for degradation. However, the creation of this complex stabilises cytosolic *β*-catenin resulting in its nuclear translocation. Within the nucleus, it induces downstream transcription of bone-related genes such as Runx2 and osteocalcin via T-cell factor/lymphoid enhancer binding factor (TEF/LEF) cofactors [25–27] (Figure 1(a)). Activation of the Wnt/*β*-catenin dependent pathway also modulates osteogenic and chondrogenic differentiation of mesenchymal stem cells as well as regulating bone mass by increasing osteoprotegerin expression thereby reducing osteoclastogenesis [28, 29]. It has been shown that sclerostin exerts its negative regulation of bone formation by binding via its central core to the extracellular domains LPR5/6 at the first *β*-propeller via an NXI motif. This prevents Wnt and FZD ligand binding [30, 31]. Fundamentally, the interference of sclerostin in the Wnt/LRP/FZD complex results in uninhibited GSK-3*β* activity and the phosphorylation of *β*-catenin leading to its subsequent degradation. Consequently, translocation into the nucleus is not actively facilitated which renders Wnt pathway gene promoters inactivated. This effectively inhibits the anabolic function of Wnt signalling for bone and as a consequence decreases bone formation [32] (Figure 1(b)).

### 3.2. Sclerostin as a Therapeutic Target

Mutations in the LRP receptors initially helped establish a link between Wnt signalling and disease and their role as positive regulators of bone formation. The LRP5 gene was initially identified as a determinant of bone mass in the 1990s. Specifically, linkage analyses studies demonstrated that the human chromosome 11q13 was associated with two extremes of low and high bone mass [33, 34]. Osteoporosis pseudoglioma syndrome, an autosomal recessive hereditary disorder (characterised by low bone mass and abnormal eye vasculature), was traced to an inactivation mutation of LRP5 [33]. In contrast, gain-of-function LRP5 mutations are linked to the autosomal dominant high bone mass (HBM) which are defined by excessive bone formation, bone thickening, and reduced risk of fracture [34, 35]. These findings were confirmed in mouse models of defective Wnt signalling. Briefly, LRP5-deficient mice manifested an osteoporotic phenotype arising from the impaired bone formation while bone resorption remained unaffected. In contrast, mice carrying an HBM mutation within the LRP5 gene display osteosclerosis [36, 37]. Several studies using Cre-loxP-technology focused on the inactivation of *β*-catenin specifically in osteoblasts and osteoclasts in an effort to understand the molecular signature of the skeletal phenotype displayed by these diseases. Essentially, these and other similar studies demonstrated that *β*-catenin deletion in cells within the osteoclast lineage resulted in increased osteoclastogenesis. In contrast, *β*-catenin deletion in mesenchymal osteoprogenitor cells resulted in the prevention of osteoblast differentiation with an associated preference for chondrogenesis [38, 39]. Importantly, *β*-catenin inactivation in fully differentiated osteoblasts and osteocytes resulted in increased osteoclastogenesis arising from the decreased osteoprotegerin while bone formation remained unaffected [29, 40, 41].

More recently it has been demonstrated that Frizzled-8 and *β*-catenin negatively regulate osteoclast differentiation independent of osteoblasts and that canonical Wnt signalling controls bone resorption by two different mechanisms [42]. The authors report that mice deficient in Frizzled-8 manifest osteopenia associated with the unhindered bone formation and increased osteoclastogenesis. Nevertheless, this phenotype was not associated with impaired osteoprotegerin production or Wnt signalling by osteoblasts. Further, *β*-catenin deletion in the osteoclast lineage confirmed the negative influence of canonical Wnt signalling on osteoclastogenesis. Here increased bone resorption was evident despite the apparently normal production of osteoprotegerin by osteoblasts being observed [42]. The reader is directed to a recent review for a more comprehensive insight into the molecular associations of LRP5 and bone formation [43]. The potential of sclerostin as a therapeutic target was soon recognised and gained momentum when animal studies with SOST knock-out mice were also found to have increased BMD over wild-type counterparts, whereas transgenic mice overexpressing SOST displayed an osteoporotic bone phenotype [18, 44]. Given the similarities to the changes observed in LRP5 mutations, the notion sclerostin functioned as an LRP5 antagonist was pursued and later confirmed. Nonetheless, it is now recognised that other interactions are also involved in the antiosteoanabolic role of sclerostin [22, 23]. More recently, Yorgan and colleagues attempted to clarify if sclerostin truly acts as a Wnt signalling antagonist by interacting with LRP5. The authors generated Col1a1-SOST mice with transgenic overexpression of sclerostin under the control of a 2.3-kb Col1a1 promoter, resulting in a low bone mass phenotype. The two mouse lines used carried different high bone mass mutations of LRP5 (LRP5 (A170V) and LRP5 (G213V)). Subsequently, the authors found that the inhibitory function of sclerostin overexpression on bone formation was not observed in LRP5 (G213V/G213V) mice and was strongly reduced in LRP5 (A170V/A170V) mice. The authors then adopted a similar approach whereby the transmembrane Wnt signalling antagonist Krm2 was overexpressed in mice. Interestingly, the antiosteoanabolic influence of the Col1a1-Krm2 transgene was not affected by either LRP5 mutation investigated [43].

Due the heterogeneous cellular make-up of bone, gaining detailed insight into the transcriptional response to Scl-mAb and the associated mechanistic nature of its modulation of Wnt signalling pathway has proved challenging. In the context of the mesenchymal stem cell osteogenic differentiation pathway, sclerostin activity has the most effect on the late osteoblast cells, inhibiting the terminal differentiation of osteoblasts and the associated mineral deposition. It has been postulated that sclerostin may function in the upregulation of small integrin-binding ligand N-glycoprotein (SIBLING) family formation. SIBLING proteins bind to newly mineralised surfaces preventing further mineralisation through acidic serine aspartate-rich motifs (ASARM peptides). It also appears that the formation of PHEX, a metalloprotease capable of inactivating ASARM peptides disruption of mineralisation, is inhibited by sclerostin too [45]. More recently, Nioi and colleagues used laser capture microdissection to assess changes in mRNA expression of specific canonical Wnt-related genes in osteoblasts, osteocytes, and bone lining cells in ovx-rat vertebrae that had undergone a single administration (100 mg/kg) of 1Scl-mAb. Samples were obtained 6, 24, 72, and 168 hours after Scl-mAb administration [46]. Microarray analyses revealed that five canonical Wnt-related genes in particular, namely, Gja1, Bglap, Twist1, Mmp2, and Wisp1, were markedly upregulated subsequent to Scl-mAb exposure, suggesting a targeted activation of canonical Wnt signalling associated genes. Notably, there was a significant upregulation of extracellular matrix-associated genes in osteoblasts, osteocytes, and bone lining cells in response to Scl-mAb treatment. This would suggest that bone anabolism is facilitated via the activation of matrix-producing osteoblasts and transition of bone lining cells to active matrix-producing osteoblasts. Interestingly, while Scl-mAb treatment has been observed in several animal and human studies to enhance bone formation and reduce bone resorption, here gene modulation of osteoclastogenesis remained unaffected. Increased osteoprotegerin is a likely candidate modulator given it is recognised as a canonical Wnt target. However, the authors did not observe any changes in expression [46]. The complexity of the mechanisms by which Scl-mAb exert their effect is an ongoing area of research and vital to a full understanding of its potential as a therapeutic target. Meanwhile, the neutralisation of sclerostin using monoclonal antibodies has been subject to numerous preclinical studies and a range of clinical trials which are addressed below.

## 4. Current Clinical Trial Data Relating to Anti-Sclerostin Monoclonal Antibody Therapy

**Figure 2:**
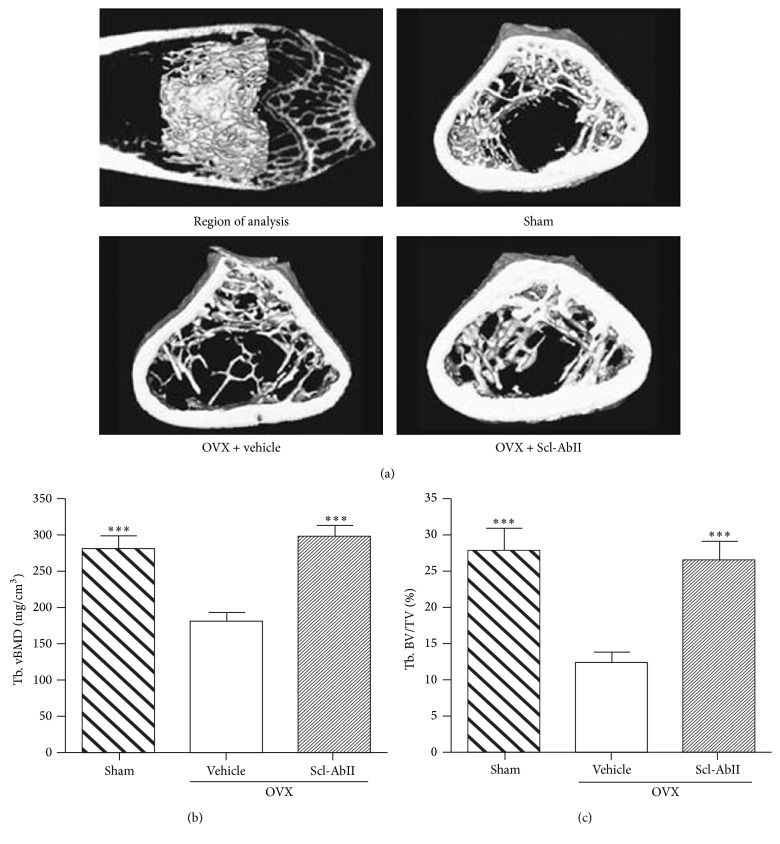
The first* in vivo* study to test anti-sclerostin antibodies for increasing bone mass involved a rodent model of postmenopausal osteoporosis. Ovariectomised (OVX) rats were treated with 25 mg/kg of sclerostin antibody twice weekly for 5 weeks while sham-operated controls (Sham) were treated with PBS. Treatment with sclerostin antibody reportedly restored trabecular bone volume and bone mineral density (BMD) to comparable levels observed in sham-operated animals. Trabecular volumetric BMD = Tb. vBMD; median trabecular bone volume = Tb. BV/TV. ^*∗∗∗*^ *p* < 0.001 versus OVX + vehicle. Reproduced with permission from Li et al. [50].

The literature on sclerostin and its inhibition with monoclonal antibodies has been well reviewed. These reviews include its discovery and development as a therapeutic target, its mode of action, and its suitability as a biochemical marker, along with more general overviews [2, 47–49]. The initial development and screening of sclerostin neutralising antibodies involved a murine IgG1 produced in a hybridoma cell line which was validated* in vitro* [50]. Due to the subsequent commercial development of the antibody, information relating to its characteristics has been limited. Nevertheless, the Eli Lilly anti-sclerostin antibody blosozumab is described as an IgG4 antibody [21] while the Novartis humanized Scl-mAb is described as an IgG2 antibody. The first* in vivo* study to test anti-sclerostin antibodies for increasing bone mass involved a rodent model of postmenopausal osteoporosis (Figure 2). Ovariectomised (ovx) rats were left untreated for one year in order to develop significant levels of bone loss. Treatment of 19-month-old ovx-rats with Scl-mAb (25 mg/kg, biweekly for 5 weeks) appeared to completely reverse the bone loss exhibited over the previous year-long period. In fact, bone strength and mass were both reportedly higher in Scl-mAb treated ovx-rats compared to non-ovx control animals [50]. More recently, it has been determined that the increases in bone formation observed due to Scl-mAb treatment in rats and primates do not negatively influence bone matrix quality even where increases in bone volume as high as 54% are noted [51]. The transition from preclinical models to human clinical trials was facilitated by a major primate study that used dosage levels and a delivery schedule intended to replicate those of early-stage human trials. This study confirmed a strong correlation between serum antibody levels and serum levels of the bone formation markers osteocalcin and P1NP and was considered to provide strong evidence that the effect of treatment was robust and reproducible [52]. A complete chronological summary of all clinical trials investigating Scl-mAb is presented in Table 1.

### 4.1. Romosozumab

Data from two phase I trials of the humanized Scl-mAb romosozumab (Amgen, formally known as AMG785) have been published. The initial first-in-human single-dose randomized study included healthy men and postmenopausal women (aged 45–59; NCT01059435). This was followed by a multidose study (NCT01825785) [4, 53]. Padhi et al. describe a randomized, double-blind, placebo-controlled, ascending-single-dose study in healthy men and postmenopausal women which received romosozumab or placebo (3 : 1) subcutaneously (0.1, 0.3, 1, 3, 5, or 10 mg/kg) or intravenously (1 or 10 mg/kg). The primary objective of the study was to establish safety and tolerability of romosozumab. Secondary objectives included the evaluation of pharmacokinetics, bone turnover markers, and bone mineral density. A total of 72 subjects were enrolled with 56 (14 placeboes, 42 romosozumab) receiving investigational product administered subcutaneously while 16 subjects (4 placeboes, 12 romosozumab) received the investigational product intravenously.

Both subcutaneous and intravenous dosing resulted in a greater-than-dose-proportional increase in romosozumab serum concentrations, with apparent clearance decreasing as dose increased. Increases in P1NP, bone-specific alkaline phosphatase (BAP) and osteocalcin (markers of bone formation) were dose-related with maximum increases from baseline of 184%, 126%, and 176%, respectively. The serum concentration of C-terminal telopeptide of type 1 collagen (CTx; bone resorption marker) concomitantly decreased in an approximate dose-dependent manner with a maximum decrease of 54% below baseline reported. As expected, BMD was also significantly enhanced with maximum increases of 5.3% in the lumbar spine and 2.8% in the hip observed. Overall, treatment was generally well-tolerated with one serious adverse event being reported for a subject that received 10 mg/kg dose. The subject experienced nonspecific hepatitis which commenced 1 day after dosing but resolved after approximately 1 week. Of the 54 subjects receiving romosozumab 11% (6/54) tested positive for binding anti–romosozumab antibodies in the highest-dose groups. Two of these subjects tested positive for neutralising antibodies (1 subject 10 mg/kg subcutaneous; 1 subject 5 mg/kg intravenous) [4]. Nonetheless, when placed in context with existing therapies, a single 10 mg/kg dose of Scl-mAb was found to produce BMD increases that were either equivalent or greater than that observed after 6 months of daily teriparatide treatment [54].

A subsequent double-blind, placebo-controlled, randomized, ascending-multiple-dose phase I trial (NCT01825785) was completed to assess the safety, tolerability, pharmacokinetics, and pharmacodynamics of romosozumab treatment for 12 weeks in 16 healthy men and 32 postmenopausal women with low bone mass [53]. Female subjects consisted of three groups. The first cohort received 6 doses of 1 or 2 mg/kg every 2 weeks (Q2W). The second group of participants received 3 doses of 2 or 3 mg/kg every four weeks (Q4W). The final group consisted of placebo treatment. The healthy male subjects received 1 mg/kg Q2W or 3 mg/kg Q4W or placebo. Romosozumab treatment in all groups was observed to increase PINP by 66–147% and lumbar bone mineral density by 4–7%, associated with a concomitant 15–50% decrease in serum CTx. Total hip BMD also increased by approximately 3% in female subjects taking romosozumab. Bone formation marker levels (P1NP, osteocalcin, and BSAP) returned to baseline 4–8 weeks subsequent to the last romosozumab dose. With the exception of increased mild injection site reactions with romosozumab, adverse events appeared balanced between treatment and placebo groups. Similarly to the single-dose study, two subjects tested positive for neutralising antibodies which reportedly had no apparent effects on the primary study objectives [53].

McClung and colleagues have recently published results from a phase II, multicentre, international, randomized, placebo-controlled, parallel-group, eight-group study, where the efficacy and safety of romosozumab over a 12-month period was evaluated in 419 postmenopausal women (55–85 years of age; NCT00896532) [55]. Subjects were randomly assigned to three groups. These comprised either subcutaneous romosozumab monthly injections (dose of 70 mg, 140 mg, or 210 mg) or every 3 months (140 mg or 210 mg), subcutaneous placebo, or an open-label active comparator-oral alendronate (70 mg weekly) or subcutaneous teriparatide (20 *μ*g daily). The primary endpoint was the percentage change in BMD in the lumbar spine at 12 months compared to baseline. Secondary endpoints included the percentage change in BMD at other sites and changes in markers of bone turnover. After 12 months, the pooled romosozumab group had significantly higher BMD compared to the pooled placebo group irrespective of the frequency of dose (monthly/every 3 months) and dose concentration (140 mg/210 mg). The largest gains were observed with the 210 mg monthly dose of romosozumab. Here, mean increases (compared to baseline) of 11.3%, 4.1%, and 3.7% in the lumbar spine, 4.1% total hip, and femoral neck were observed, respectively. Furthermore, the increases in BMD observed were significantly greater than the alendronate (4.1% increase in BMD in the lumbar spine) and teriparatide groups (7.1% increase in BMD in the lumbar spine).

Interestingly, serum levels of bone formation markers in this trial were reported to increase transiently. This contrasts the robust and reproducible increases in serum bone formation markers reported subsequent to romosozumab treatment in the iconic primate study [52]. McClung and colleagues observed that increases in serum bone formation markers occurred as soon as 1-week after administration with peak levels reported after 1 month. However, levels returned or fell below baseline values within 2–9 months (dependent on dose and marker evaluated). All romosozumab groups demonstrated a decrease from baseline in the level of CTx initially, with the largest median decrease evident after one week. Furthermore, subjects receiving monthly doses of romosozumab maintained levels of CTx below baseline after 12 months [55].

The incidence of serious adverse events in the pooled romosozumab group was 7% which was comparable to the placebo (14%), alendronate (8%), and teriparatide (9%) groups. The 210 mg (administered every 4 weeks) romosozumab group had 10% (5 subjects of 51) occurrence of serious adverse events. These included breast cancer, chronic obstructive pulmonary disease, noncardiac chest pain, wrist fracture, and begin renal oncocytoma. However, no serious adverse event was reported by more than 1 participant in any group and none were considered by the investigator to be treatment-related. As observed in the previous phase I studies, binding antibodies were identified in 20% of subjects receiving romosozumab, with 3% of these demonstrating* in vitro* neutralising activities. Despite their occurrence, there was no apparent effect on the occurrence of adverse events, pharmacokinetics, or pharmacodynamics [55]. Overall, given the significant and prompt increases in BMD subsequent to romosozumab treatment compared to alendronate and teriparatide, this study further supported the application of romosozumab in the treatment of osteoporosis.

More recently, this trial was extended to include an additional 12-month treatment period under similar study conditions as outlined above. Although only currently available in the abstract form, the increases in BMD in the lumbar spine and total hip observed during the first 12 months of treatment were further increased with the additional 12-month romosozumab. The largest gains in BMD were again observed with the 210 mg monthly dose (15.7% lumbar spine BMD; 6.0% total hip BMD). This study was extended to include a one-year double-blind extension phase where eligible subjects (women receiving 210 mg romosozumab dose) were rerandomized 1 : 1 within their original treatment group to placebo or denosumab 60 mg once every six months (*n* = 260). Women receiving romosozumab 210 mg monthly who then transitioned to treatment with denosumab (targets RANKL) after 12 months continued to accrue BMD at a rate similar to that observed during the second year of treatment with romosozumab. However, subjects that transitioned to placebo demonstrated a return in BMD towards pretreatment levels. Furthermore, in subjects where romosozumab treatment was discontinued after 2 years, a decrease in BMD towards baseline levels and the return of bone formation marker P1NP to pretreatment levels were observed [56].

Another ongoing study is assessing the impact of romosozumab treatment compared to teriparatide treatment on vertebral mass, thickness, and density. Although not published, initial results conveyed by Amgen suggest that treatment with romosozumab produced an increase in cortical thickness of 11.2% compared to 5.6% with teriparatide. Furthermore, romosozumab induced an increase of 22.2% in trabecular BMD compared to a 17.4% increase with teriparatide. In contrast, a reduction of 4.3% was reported in the placebo group (NCT01796301). A phase I trial assessing changes in baseline lumbar spine BMD after the transition from alendronate to romosozumab for 3 months has recently been completed with results pending (NCT01588509). It would certainly appear that romosozumab has great promise in the treatment of osteoporosis and based on these results it would appear that market approval may be foreseeable. Less clear at the moment, however, are efficacy issues regarding long-term treatment (i.e., in excess of 2 years as a decrease to baseline is observed after discontinuation) and safety concerns relating to potential long-term treatment schedules. For the moment, at least, it would appear that romosozumab would be highly beneficial in the short-term and is compatible with antiresorptive therapies.

### 4.2. Blosozumab

Two phase I trials of blosozumab (Eli Lilly) were undertaken. The first study included a single-dose, dose escalation study (7.5 to 750 mg i.v.; NCT01742078). This was followed by a multicentre safety and tolerability study of multiple-dose administration (Q2W, Q4W; NCT01742091) [57]. Participants were otherwise healthy postmenopausal women ranging in age within 45–70 years of age (single-dose) and 45–80 years of age (multidose). Both studies were subject and investigator blinded and placebo-controlled. Overall, the treatment was generally well-tolerated. However, antibodies to the drug were discovered upon screening (23% of patients in the single-dose study; 36% in the multidose study). Nonetheless, titres were low and their occurrence did not appear to impact pharmacodynamics. Furthermore, neutralising antibodies were not detected. Dose-dependent increases in drug serum levels and bone formation markers P1NP and BAP and osteocalcin along with a decrease in CTx were recorded. Treatment also resulted in increased BMD in the lumbar spine with a maximum increase above baseline of 3.41% after a single dose and 7.71% after multiple doses after 85 days (compared to 5.3% and 7.2%, resp., for single and multiple-dose administration of AMG 785).

These positive clinical results were followed in a more recently randomized, double-blind, placebo-controlled multicentre phase 2 clinical trial of blosozumab comprising 120 postmenopausal women (45–85 years) with low bone mineral density (lumbar spine BMD T-score of −2.0 to −3.5; NCT01144377). The study encompassed a 1-year treatment period with a 3-month follow-up period once treatment ended. The primary objective was to evaluate the dose-response of 180 mg (every 2 or 4 weeks) to 210 mg (Q2W) (compared to 70 mg–210 mg in the phase two trial of romosozumab) of blosozumab on lumbar spine BMD [21].

In terms of efficacy, mean increases in BMD in the lumbar spine were statistically significant for all treatment groups. A maximum increase in the lumbar BMD of 17.7% above baseline in the group receiving 270 mg every two weeks was reported. In the 3-month follow-up study, the BMD in all dosage groups declined when blosozumab treatment ceased. Levels of P1NP peaked at week 4 and remained significantly above baseline for 24 weeks and returning towards baseline levels by the end of the study. Levels of osteocalcin and bone alkaline phosphatase also increased with blosozumab treatment and returned towards baseline by the end of the study. The bone resorption marker CTx levels decreased to less than placebo within two weeks and were similar to placebo at 12 weeks and reduced compared to placebo at 52 weeks [21].

Mild injection site reactions were reported by up to 40% of patients receiving blosozumab; otherwise, adverse events were similar between treatment and placebo groups. Four women taking blosozumab were diagnosed with breast cancer within 3 months to 1 year of the beginning of the trial; however, examination suggested these were likely to be preexisting tumours. Anti-blosozumab antibodies were found in 35% of those treated with one patient developing neutralising antibodies that resulted in greatly reduced efficacy from treatment [21].

A one-year follow-up study assessing the effects of discontinuing blosozumab treatment in the phase II patient cohort has recently been published [58]. No serious adverse effects after discontinuation of the treatment were observed. With the discontinuation of treatment, a decline in BMD in both the femoral neck and the lumbar spine was observed in all blosozumab treatment groups, which continued through the 1-year follow-up period. However, these remained significantly higher than placebo groups. Moreover, serum biochemical formation and resorption markers did not differ significantly between previously treated blosozumab and placebo groups. While in the initial phase II trial approximately 35% of blosozumab treated patients presented with antitreatment antibodies, these were seen to decline with discontinuation of the treatment [58].

Overall, the results from the phase two trial were encouraging. While no serious safety issues were raised, John Lechleiter the Chairman of the Board, President, and Chief Executive Officer of Eli Lilly explained that phase III trials proposed for 2014 were delayed due to higher than desirable levels of injection site reactions. This led to the reassessment of the formulation used in their phase II trials before moving forward. The occurrence of injection site reactions appears to be a common occurrence subsequent to antisclerostin treatment for all humanized Scl-mAb tested and is currently under investigation by both Amgen and Eli Lilly.

### 4.3. BPS804

Three BPS804 phase II trials to treat postmenopausal women with low BMD (NCT01406548), osteogenesis imperfecta (NCT01417091), and hypophosphatasia (NCT01406977) have been completed. To date, no data has been published and there is no information available in relation to plans for phase III trials. An additional phase II trial to investigate the safety and tolerability of BPS804 in patients with late-stage chronic kidney disease (NCT01806610) has since been withdrawn prior to initiation of patient enrolment. Not surprisingly, therefore, BPS804 does not currently feature in the clinical pipeline reports for 2014–2018 published by Novartis [59]. Nevertheless, the company's interest in fundamental research pertaining to the mechanistic actions of sclerostin in bone repair persists [60–62].

### 4.4. Combined Treatments

The combined therapy approach of Scl-mAb antibodies followed by ongoing use of antiresorptive drugs to maintain enhanced bone formation is another emerging area of investigation. The use of alendronate in relation to pretreatment and cotreatment with antisclerostin therapy has been assessed in animal studies with encouraging results [63]. This stands in contrast to the currently approved anabolic treatment with human parathyroid hormone (PTH). In this case, it was hypothesised that cotreatment with alendronate would enhance the anabolic qualities of PTH. However, the combined treatment was actually found to have a reduced anabolic effect in clinical studies [64, 65].

The cotreatment of anti-tumour necrosis factor (anti-TNF) along with anti-sclerostin antibodies in human TNF transgenic mice has recently been shown to be more effective than either treatment administered alone. Specifically, cotreatment of mice with anti-TNF and Scl-mAb was found to be effective in repairing cortical lesions, cartilage destruction, and preventing proteoglycan loss. These results were an improvement compared to anti-TNF or Scl-mAb alone which appeared to prevent further disease progression but did not support tissue repair. For instance, in this model, the combination of anti-TNF and Scl-mAb treatment was observed to prevent cortical and trabecular bone loss (an increase of 34% compared to baseline) and restored vertebral bone to levels observed in nonarthritic wild-type mice. The combination of anti-TNF and Scl-mAb significantly decreased arthritic bone erosion compared to baseline as well as reducing osteoclast number. Interestingly, compared to baseline levels, Scl-mAb alone or in combination with anti-TNF significantly increased cartilage thickness, area, and proteoglycan content. Interestingly, the largest increases were observed in the cotreatment groups [43].

This combination of anti-inflammatory treatment with bone enhancing antibodies could also have potential to aid the treatment of decreased BMD due to colitis. The option of alternating anabolic and antiresorptive therapies has also been the focus of several recent clinical trials relating to romosozumab treatment and is highlighted in Section 4.1.

## 5. Emerging Alternative Clinical Applications for Sclerostin mAb Therapy

### 5.1. Fracture Healing

The efficacy of anti-sclerostin antibody therapy in the treatment of fragility fractures has been tested in rodent and nonhuman primate preclinical models [66–69]. More recently, two phase II clinical trials (NCT00907296) (NCT01081678) have also investigated this application. In all rodent experimental models (rat diaphyseal defect, rat closed mid-diaphyseal femoral fracture, rat femoral osteotomy, and murine femoral osteotomy), subcutaneous administration of 25 mg/kg of Scl-mAb twice weekly for varying time courses confirmed enhanced bone healing with significant increases in bone formation, mass, and strength [66–69]. Furthermore, Cui and colleagues report that the size of the repair callus in Scl-mAb treated mice with osteotomies was increased as early as 2 weeks after treatment compared to controls. The Scl-mAb treated group was associated with a faster fracture union by week 6 and significantly higher maximal loading capacity [66]. Similarly, Suen and colleagues report an increase in fracture callus size of 23–30% at 3, 6, and 9 weeks after Scl-mAb administration compared to vehicle controls. Histologically more bony tissue and less cartilage tissue were observed in fracture calluses across all time points in the Scl-mAb treated groups. Moreover, the proportion of mature callus tissue was significantly greater with the Scl-mAb treatment at weeks 6 and 9. This was reflected by the significant increases in total bone volume (26–33%) and high-density bone volume (38–42%) compared to vehicle groups. This was also associated with more rapid progression of fracture repair supported histologically. The Scl-mAb treatment also resulted in faster mineral deposition compared with vehicle controls. Furthermore, Scl-mAb increased the rate of new bone formation in both the total callus (41%) and the periosteal callus subregion (42%) at 9 weeks. These positive effects of Scl-mAb treatment also translated to mechanical outcomes. Specifically, a significantly higher load in Scl-mAb treated groups was reported at weeks 6 (98%) and 9 (53%) after fracture compared to controls [67]. Similarly, Scl-mAb treatment resulted in significant increases in callus stiffness and energy to failure. The positive impact of Scl-mAb treatment in fracture repair setting is also reflected elsewhere [70].

More recently, Yee and colleagues have demonstrated enhanced bone formation using Scl-mAb in an early on-set Type I diabetic mouse fracture model. Here the authors report that Scl-mAb treatment rescued impaired osteogenesis and marrow adiposity that is associated with the diabetic phenotype. Moreover, in uninjured bone, the positive effect of Scl-mAb on bone formation persisted for up to 3 weeks after discontinuation of the biweekly (25 mg/kg) treatment [71]. Despite the promise of Scl-mAb treatment in fracture repair, the reparative effect does not appear to reproducibly extend to nonunion fractures [72, 73]. For example, Alaee et al. have recently demonstrated that treatment with Scl-mAb can enhance bone repair in the surrounding bone of a rat femoral critical-sized bone defect but does not possess osteoinductive activity to heal it [73]. Thus, it seems that Scl-mAb is effective in enhancing bone formation of preexisting and regenerating bone tissue but does not appear to be an osteoinductive agent. This appears to be supported by a recent study that has shown that endochondral bone formation persists in fractures lacking sclerostin (SOST −/− mice) while fibrocartilage callus removal was enhanced. The resultant bony calluses displayed increased bone fraction and strength [62].

Ominsky et al. examined Scl-mAb treatment for the repair of bilateral fibular osteotomies in cynomolgus monkeys [68]. Treatment consisted of 30 mg/kg of anti-sclerostin antibody twice weekly for 10 weeks. The therapy led to an increase in serum levels of the bone formation markers osteocalcin and procollagen 1 N-terminal propeptide (P1NP) and an associated increase in bone formation. After just 7 weeks, fractures in the vehicle and Scl-mAb groups healed to 27% and 48% of the mean peak load of the intact contralateral femurs in the vehicle group. In addition, Scl-mAb administration significantly improved the rate by which the majority of intact strength was achieved compared to vehicle-treated groups. The rate to union was also markedly improved with Scl-mAb treatment. Specifically, 9/10 in the latter group achieved union compared to 4/9 fractures in the vehicle-treated group. Histologically, there was a significant increase in bone formation within the fracture area in Scl-mAb treated groups. This was associated with reductions in the persistence of a cartilaginous callus within this group. The smaller fracture gaps observed within the Scl-mAb groups also displayed less fibrovascular tissue compared to vehicle-treated groups, although this difference was not statistically significant. At the fracture site, Scl-mAb treatment resulted in a 27% increase in mature bone callus formation associated with a 30% increase in bone mineral content. Furthermore, Scl-mAb produced a 48% greater mean torsional stiffness and 32% greater peak torque compared to vehicle controls [68].

Both phase II trials assessing Scl-mAb in fracture repair (NCT00907296 and NCT01081678) directed by Amgen have submitted requests in May 2013 and January 2014, respectively, to delay publication of the trial results. At the time of writing, neither blosozumab (Eli Lilly) nor BPS-804 (Novartis) appears to be undergoing investigation at clinical trial. Therefore, to date, the efficacy of Scl-mAb therapy in the application of fracture repair has not been established in human trials. Furthermore, a press release early in 2013 confirmed that Amgen would not be pursuing Scl-mAb treatment for fracture healing into phase III trials [74]. Sclerostin levels are greatly increased in the early stages of fracture healing which may add to the complexity of providing effective neutralisation to enhance fracture repair [75]. Fracture repair and related aftercare currently account for a staggering 95% [12] of the cost of all osteoporosis treatments. The lack of Scl-mAb therapy in this area will see patients remain restricted to the prophylactic treatment options to reduce fracture risk for the foreseeable future.

### 5.2. Implant Fixation

The secure fixation of bone implants presents another complication in the treatment of osteoporosis. High failure rates are reported for total hip replacement due to the weakness of the surrounding bone. The anchorage of screws and pins used in fracture repair can also be compromised due to poor bone quality [10, 11]. Preclinical studies have demonstrated efficacy in significantly enhancing bone fraction volume adjacent to the implant subsequent to Scl-mAb administration. Treatment has also been shown to increase peak pull-out forces [76, 77]. Moreover, screws inserted postmortem also showed increased pull-out force in the treated group. This would appear to imply a general rather than injury specific response to antibody treatment [76]. Moreover, Scl-mAb administration has demonstrated promise in preventing periprosthetic osteolysis and aseptic loosening in a preclinical study [78]. More recently, in a severe model of osteoporosis (approximately 78% trabecular bone loss at the time of implantation) systemic Scl-mAb administration has also proven effective in stimulating osseointegration via enhanced bone formation. Here, the authors report improved trabecular bone volume and architecture as well as decreased bone resorption subsequent to Scl-mAb treatment [79].

### 5.3. Treatment of Other Diseases Impacting Bone

In addition to the treatment of osteoporosis, antisclerostin therapy has been explored as a treatment for other conditions causing reduced bone mineral density. Preclinical animal studies encompassing periodontitis, colitis, rheumatoid arthritis, osteoarthritis, bone health complications of diabetes mellitus, chronic kidney disease, and osteogenesis imperfecta (OI) have all been encouraging [80–85]. However, there appears to be some contradiction between studies possibly resulting from the different animal models used or the disease stage in which Scl-mAb were administered. For instance, treatment of severe OI with Scl-mAb had limited success in enhancing trabecular bone volume and cortical thickness in growing mice (4 weeks). In contrast, treatment failed to have any significant impact in adult OI mice (20 weeks). Furthermore, neither growing nor adult OI mice displayed treatment-associated alterations in bone remodelling serum markers compared to wild-type controls [86]. In contrast, moderately severe OI in the Brtl/+ mouse model of OI responded positively to Scl-mAb treatment. Here, treatment resulted in stimulated osteoblast-mediated bone formation leading to increases in bone mass and reduced long-bone fragility [83, 86]. Bisphosphonates are currently used in the treatment of OI and have been shown to lower the risk of fracture in some cases [87, 88]. While generally well-tolerated, there are, however, unresolved issues relating to the use of bisphosphonates in OI. These include issues relating to long-term retention, clinical reproducibility of lowering fracture risk, functional benefit, and potential childhood bisphosphonate toxicity [89–92]. Moreover, other anabolic treatments such as growth hormone and teriparatide have provided mixed outcomes. For instance, prolonged use of teriparatide may be potentially associated with the development of osteosarcoma [93, 94]. Given these issues and the observed anabolic effect of Scl-mAb in osteoporosis and fracture repair, it is speculated that this therapy may be beneficial in the treatment of OI. Specifically, it is thought that Scl-mAb treatment may benefit paediatric OI patients in particular by increasing bone mass and mechanical strength and reducing the risk of fracture. A 2-week treatment course of Scl-mAb was found to produce increases in bone mass and mechanical strength in the well-established Brtl/+ mouse model of moderately severe Type IV OI. However, more extensive studies are required to fully establish the potential of Scl-mAb in OI [83], Moreover, given that it is likely that Scl-mAb therapy will need to be administered as an adjunctive therapy (see Section 7), the long-term benefits in OI need to be addressed.

Recently, Chen et al. combined anti-TNF t with Scl-mAb treatment in a mouse model of rheumatoid arthritis. The combination therapy was found to contribute to repair of damaged articular cartilage and eroded bone, whereas either treatment alone only prevented progression of disease symptoms [81]. Chondrocytes reportedly secrete sclerostin. However, a recent study has suggested that pharmacological inhibition of sclerostin does not impact articular cartilage remodelling; therefore, an explanation for the positive outcomes described above is yet to be fully elucidated [81, 95].

Periodontitis, a destructive disease of the tooth-supporting structures ultimately resulting in tooth loss, is another area which may potentially benefit from Scl-mAb therapy to repair large osseous defects. Recent evidence suggests that sclerostin (as well as DKK-1) is significantly increased in the gingival tissue and the serum of chronic periodontitis patients. These findings suggest a possible molecular link between sclerostin and periodontal disease [96]. Using a variety of methods including lineage tracing and knock-out models, it has recently been shown that activation of the Wnt-canonical pathway via Scl-mAb treatment results in cementum and alveolar bone regeneration [85, 97, 98]. Taut and colleagues recently investigated the effect of administering 25 mg/kg Scl-mAb administration delivered twice weekly for a therapeutic duration of 3 and 6 weeks in an experimental periodontal rat model. Notably, after 6 weeks, the Scl-mAb treated group displayed reversed ligature-induced bone loss. This outcome was also associated with a significant increase in bone volume and tissue mineral density compared to vehicle-treated controls. Interestingly, local administration of Scl-mAb displayed limited effect on volumetric alveolar bone healing. Alveolar bone loss was, however, significantly improved after 6 but not 3 weeks subsequent to systemic Scl-mAb treatment compared to vehicle-treated groups. Bone densitometry scanning was also performed on femora to assess off-site skeletal responses to Scl-mAb administration. The femora of all treatment groups demonstrated significant increases in bone mineral density at both 3- and 6-week time points. Bone formation markers were also increased after Scl-mAb administration which translated to the restoration of lost bone microarchitecture, volume, and density to levels comparable to intact control after 6 weeks. Interestingly, the application of Scl-mAb as a preventative measure to alveolar bone loss (administered when sutures were initially placed and while at the site of defect) only resulted in a slight and nonsignificant increase in bone mass after 2 and 4 weeks. These findings highlight the suitability of Scl-mAb as an adjunctive therapy in this context [99].

Chen and coworkers also studied the effect of administering 25 mg/kg vehicle or Scl-mAb subcutaneously twice weekly for 6 weeks in ovariectomised rats with experimental periodontitis (ligature). The authors noted that the administration of Scl-mAb leads to significant increases in the bone mineral apposition rate in ovx-rats compared to controls. The authors attribute this finding to increased bone formation and decreased bone resorption evident from significant increases in osteocalcin and osteoprotegerin and serum tartrate-resistant acid phosphatase and CTx, respectively [100]. It is worth considering that current osteoconductive agents, such as bone substitutes used in this application, are clinically unpredictable in their consistency to form bone [101]. Consequently, in an effort to recapitulate the complex biological events involved in wound healing and repair, a multiphasic approach is becoming necessary [102]. As noted previously, current evidence would suggest that Scl-mAb possesses osteoconductive rather than osteoinductive properties [62, 73]. Therefore, it remains to be seen if (a) Scl-mAb is superior to current available osteoconductive agents such as commercial bone substitutes/grafts at producing more clinically consistent bone regeneration and (b) if Scl-mAb treatment, either systemically applied or locally administered via a multiphase scaffold or current commercial grafts, could provide a more robust outcome of targeted bone repair.

Growing evidence suggests that circulating Wnt signalling inhibitors such as DKK-1 and sclerostin may crucially contribute to the pathogenesis of chronic kidney disease-associated bone mineral disorder (CKD-MBD). Consequently, these have been explored as therapeutic candidates. Serum sclerostin levels have been shown to increase with CKD-MBD progression and may be potentially linked with cardiovascular events observed in this patient population [103, 104]. In addition to its prominent role in bone remodelling, the Wnt pathway and several of its inhibitors including sclerostin are increasingly associated with the occurrence of extraosseous mineralisation. This occurrence is similar to that observed in cardiovascular calcification and calciphylaxis, a rare life-threatening condition which manifests predominately in patients with CKD or end-stage renal disease [105, 106]. The biological similarities of vascular calcification and bone formation are gaining increased attention and it is believed that BMP-2, in particular, may be a vital link. Specifically, BMP-2 function can be inhibited by active matrix Gla protein (MGP), the activator of which is vitamin K dependent [107]. Moreover, vitamin K antagonist usage has been implicated as an independent risk factor for the development of calciphylaxis [108]. Sclerostin also antagonizes the effect of BMP-2 indirectly via inhibition of the Wnt/*β*-catenin signalling pathway. This may help explain the biological link between circulating serum sclerostin levels and its link to cardiovascular events observed in recent studies [109]. Nevertheless, the potential of sclerostin as a biomarker in this context requires additional confirmation. Preliminary evidence in a cross-sectional multislice computed tomography scanning study of 67 chronic haemodialysis patients suggests that increased sclerostin expression was colocalised at the sites of calcifying aortic heart valve disease [105]. Others have implicated increased serum sclerostin levels in CKD patients to be statistically correlated with inflammation, vascular lesions, uremia, and potentially mortality [110]. In contrast, others have found a contradictory correlation whereby higher levels of serum sclerostin were associated with improved survival in prevalent haemodialysis patients [111]. Moreover, in a recent prospective study of 673 incident dialysis patients, increased serum levels of sclerostin were associated with lower short-term (up to 18 months) cardiovascular (hazard ratio 0.29; 95% CI 0.13–0.62) and all-cause mortality (hazard ration 0.39; 95% CI 0.22–0.68). This trend became less significant over a 4-year follow-up period [112]. At this point, there is no clinical data to underline the therapeutic efficacy of Scl-mAb in CKD-MBD. However, recent preclinical animal studies have reported enhanced bone volume and mineralisation with Scl-mAb treatment specifically when PTH levels are low [113, 114].

## 6. Ongoing Developments: Antibody Fragments and Single Chain Antibodies 

Good tolerability has generally been reported subsequent to Scl-mAb administration. However, the development of neutralising antibodies to antisclerostin treatment has been reported including one case which seriously reduced the efficacy of treatment [4, 21, 53, 55]. Moreover, the potential immunogenicity of full-size monoclonal antibodies has been raised as a general safety concern [95]. Ultimately, ongoing phase III trials of full-size sclerostin neutralising antibodies will determine if immunogenicity is sufficient to cause concern. Nevertheless, while isolated, these events have contributed to the ongoing development of smaller antibody fragments [95, 115, 116]. For instance, Roudier et al. have shown the sclerostin-neutralising activity of sclerostin antibody fragment (Scl-Fab; MW 48 KD) to be as effective as that of the parent Scl-mAb IgG in the rat medial meniscus tear model of osteoarthritis. Moreover, the sclerostin single chain fragments (Scl-scFv; 35  KD) were observed to increase bone density, enhance bone formation, and improve bone microstructure in a rat preclinical model of osteoporosis. However, while Scl-scFv demonstrated high specificity and affinity, the authors note it had lower stability than the full-sized antibody [116].

## 7. Clinical Considerations

The route to market approval for any novel therapeutic agent is never easy and in the case of antisclerostin treatment the stakes are incredibly high. Years of research stand in the balance along with an estimated $99 million in global sales annually if market approval is secured [117]. While encouraging results from both preclinical and clinical studies have grabbed attention, a relatively small number of clinical safety and efficacy considerations have emerged. One serious adverse event has been reported in the first clinical trial of AMG785 (NCT01059435). Here, one subject developed severe nonspecific hepatitis which began one day after receiving 10 mg/kg of AMG785 (romosozumab). The hepatitis was resolved after 26 days, and additional cases of hepatitis have not been reported in subsequent trials to date [4].

As previously mentioned, the study of sclerosteosis and van Buchem disease uncovered the role of sclerostin in the modulation of the Wnt/*β*-catenin dependent pathway. As a precaution, therefore, prominent health issues relating to both conditions were investigated. While heterozygous carriers of sclerosteosis have higher than normal BMD, they are otherwise considered clinically normal [118]. However, bone overgrowth in the skull that constrains the cranial nerves and induces facial palsy can occur. Deafness can also arise due to impaired movement of middle ear ossicles [14]. A phase II clinical trial of blosozumab tested both clinical manifestations. There was no indication of a disproportionate increase in the bone mineral content of the skull. Results from brainstem auditory evoked potential tests to determine any negative impact on nerve function and hearing were also considered unremarkable by a blinded clinician [21].

The Wnt/*β*-catenin signalling is involved in a wide variety of developmental and adult tissue processes. Unsurprisingly, therefore, dysregulation of this integral pathway is associated with a multitude of diseases including cancer, fibrosis, and neurodegeneration [119, 120]. A commercial therapeutic specifically targeting the Wnt-pathway is not currently available. With market approval of Scl-mAb fast approaching, it is worth considering potential clinical implications [121]. Recent work has shown that constitutive activation of *β*-catenin can potentially negatively impact bone. Jia and colleagues constitutively activated *β*-catenin using Catnb+/lox(exon 3) mice which were crossed with mice expressing a tamoxifen-inducible procollagen I Cre-ER promoter. They demonstrate that constitutively activated *β*-catenin resulted in an excessive bone volume within the vertebral column in both early postnatal (3 days) and mature tissue (up to 7 months postnatally) [122]. However, the authors noted that early stabilisation of *β*-catenin essentially slowed linear bone growth within the vertebrae by retarding growth plate maturation. This resulted in shorter stature mice. Moreover, histologically the excessive newly formed bone appeared immature and occurred primarily adjacent to the growth plate. In contrast, late-stage *β*-catenin stabilisation appeared to not affect bone maturity or distribution [122].

More recently, it has also been shown that timely suppression of the Wnt/*β*-catenin pathway is required for osteocytic differentiation to occur adequately. In this study, *β*-catenin was constitutively activated in osteocytes by crossing Catnb+/lox(exon 3) mice with dentin matrix protein 1-Cre transgenic mice [123]. Interestingly, stabilisation of *β*-catenin in osteocytes was observed to substantially increase cancellous bone mass. However, the activation was noted to have severe adverse effects on bone strength and bone growth. Specifically, impaired mineralisation resulted in thinner and more porous cortical bone. Similar to Jia et al. [122] mice were shorter and presented with impaired linear growth of the long bones [123]. The integral role of Wnt/*β*-catenin signalling in development and disease, as well as the effects described in recent studies, should certainly be seriously considered prior to the adoption of an application involving its therapeutic interference.

Direct comparisons between humanized Scl-mAb are currently unavailable. However, a curious difference between a recent phase II blosozumab study and a prominent primate study assessing romosozumab is of potential interest [21, 52]. Clearly, comparison of a year-long human clinical trial and a 2-month long primate study assessing structurally different humanized forms of Scl-mAb is anecdotal. Nevertheless, in the absence of an explanation, it is worth briefly discussing. In the human clinical trial using blosozumab, P1NP levels increased promptly during the first 4 weeks of treatment. Concurrently, the serum concentration of the bone resorption marker CTx rapidly decreased to a concentration below that noted for the placebo group within the first 2 weeks of treatment. By the end of the study, P1NP levels were comparable to pretreatment levels while CTx remained reduced [21].

**Figure 3:**
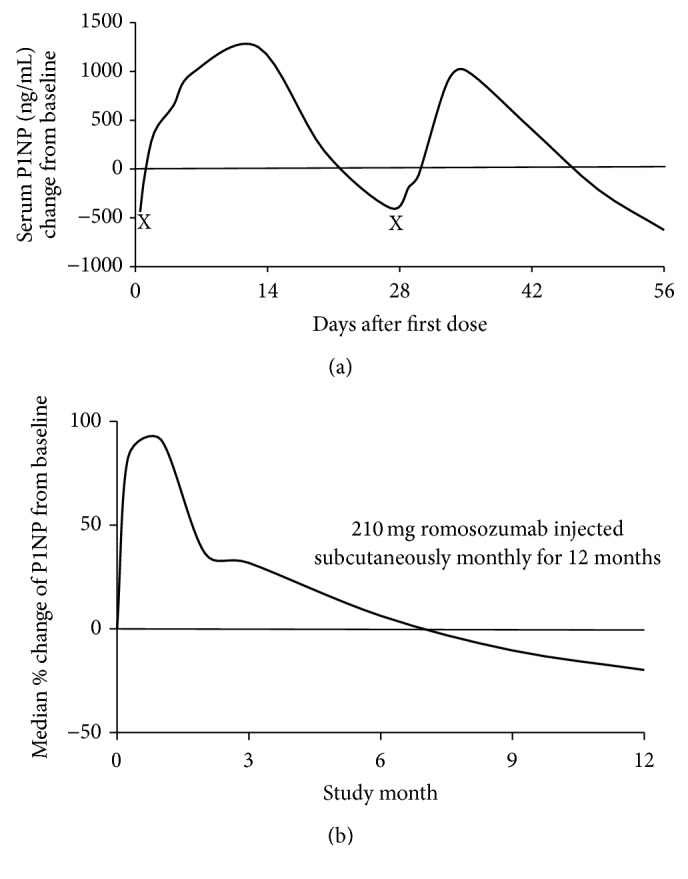
Change in serum concentrations of P1NP after multiple doses of anti-sclerostin antibodies in cynomolgus monkeys (a) and humans (b). (a) After 30 mg/kg, subcutaneous dose of romosozumab P1NP levels (a marker of bone formation) increases in cynomolgus monkeys. Subsequently, P1NP levels decline before increasing subsequent to the second dose of romosozumab on day 29 (day of administration delineated by “X”). (b) This sharply contrasts with median levels of P1NP observed in human subjects after monthly doses of 210 mg (total monthly dose) of blosozumab over a 12-month period. Here a sharp increase in P1NP is observed after initial administration which peaks after the second subcutaneous injection of romosozumab at 1 month. Despite subsequent monthly doses, P1NP levels continue to decline and were observed to fall below baseline levels after 7 months, where levels remained until 12 months (demarcated the end of the study) [images adapted from [52] (a) and [55] (b)].

The authors suggest that the return of P1NP towards baseline levels in the later stage of treatment (after week 25) may be a result of the initial increase in bone formation reducing stresses and strains on the skeletal system. Consequently, the positive signal to induce bone formation is dampened. However, this was not apparent in primate studies (Figure 3) [52]. Moreover, signalling molecules such as DKK-1 may be actively involved by reducing bone formation via negative counterregulation [124]. Importantly, the observations that blosozumab increases bone formation, decreases bone resorption, and increases spine and total hip BMD are consistent with the recently published romosozumab phase II trial comprising over 400 subjects [55]. The clinical implications, if any, in the discrepancy of serum P1NP levels in human and nonhuman primate studies remain unclear. Similarly, the causes relating to the transient changes in biochemical markers of bone formation during blosozumab treatment have yet to be elucidated.

Some clarification may be sought in a recent study which assessed Scl-mAb (25 mg/kg) treatment administered once weekly for 6 or 26 weeks in ovx-rats [125]. It should be noted that this study did not involve the blosozumab antibody. Nevertheless, the study remains relevant given its investigation of the mechanistic effect on longer-term responses of osteoclasts, osteoblasts, and osteocytes subsequent to Scl-mAb treatment. Specifically, the authors observed that changes observed in bone resorption and formation at 6 weeks in ovx-Scl-mAb treated rats largely did not persist after 26 weeks. For instance, at 6-week Scl-mAb treatment induced a net reduction in bone resorption as evident from decreases in serum TRACP-5b and a reduction in the* ex vivo* capacity of marrow cells to differentiate into osteoclasts. Moreover, an 80% reduction in surface erosion within the vertebrae and trabecular and tibial endocortical surfaces compared to ovx-vehicle samples was noted. At 26 weeks, however, the serum TRACP-5b and reduced* ex vivo* osteoclastic differentiation capacity of marrow cells were no longer evident. The observation that eroded surfaces remained over 80% lower in control groups nonetheless persisted.

Interestingly, at this time, the authors did not observe changes in RANKL or osteoprotegerin expression. As expected, Scl-mAb treatment induced significant increases in bone formation reflected by augmentations in serum P1NP and osteocalcin. Additionally, increases in the bone formation rate within trabecular, endocortical, and periosteal regions were observed. By week 26, however, only changes within the endocortical regions persevered while increases in skeletal mRNA of osteocytic genes were evident. In particular, SOST appeared to be markedly induced within the tibia and vertebral bone tissue [125]. One can cautiously speculate that the net increases in bone volume caused by the constitutive activation of Wnt-canonical signalling via Scl-mAb treatment may be synergistically counterregulated via increased osteocytic mediated sclerostin expression. Although these findings cannot be directly inferred to human outcomes, they may provide some insight as to the interstudy differences noted above. The potential for counterregulatory signalling to reduce bone formation will require further study to address its impact on longer-term treatments. To date, however, all published studies have demonstrated efficacy despite these observations. Taken together, these observations may better inform Scl-mAb treatment schedules. For now, the combination of Scl-mAb in conjunction with antiresorptive agents has demonstrated the most efficacy at trial. Therefore, it is likely that this approach will be the regimen that receives regulatory approval.

Generally, the safety profile of Scl-mAb treatment is positive, especially when compared to competitor osteoporotic therapeutics in development. For instance, the cathepsin K inhibitor MK 0822 (Merck, Odanacatib*™*) appears to increase, albeit not to statistically significant levels, the occurrence of atrial fibrillation and stroke in treated versus placebo groups [126]. These safety concerns with MK 0822 were only brought to light with the release of data from a phase III trial. Similarly, the full extent of the safety profiles of romosozumab and blosozumab cannot be fully determined from the comparatively small phase II studies.

## 8. Summary and Outlook

Compared to current anabolic market competitors such as teriparatide which is limited to 2 years' lifetime use, the substantial improvements in bone formation and microarchitecture subsequent to Scl-mAb treatment have been unrivalled. Consequently, the results of clinical studies demonstrating the therapeutic potential of romosozumab in phase III trials are eagerly awaited. While the clinical data relating to efficacy appears clear, there remain significant questions relating to optimal treatment interval. For instance, given its apparent superiority to increase BMD, should Scl-mAb be the first line of treatment for osteoporotic patients? However, it remains to be established if Scl-mAb can be used for a long term in a cyclical manner either alone or in a complementary manner with antiresorptive options. Many studies have already been performed following Scl-mAb therapy with an antiremodelling agent. It is likely, therefore, that this regimen will receive regulatory approval. This approach may help address questions attesting to the tolerability of Scl-mAb and whether the emergence of neutralising antibodies may become more of an issue with increased exposure.

The emergence and clinical success of Scl-mAb have reinvigorated the therapeutic market for osteoporosis. Despite the many questions that remain, preclinical studies and published clinical trial results would imply that Scl-mAb will emerge as a dominant first-line treatment in the management of osteoporosis. Moreover, Scl-mAb is set to become a valuable tool in the development of international strategies to address the rise of osteoporosis and osteoporotic fracture management on a global scale.

## Figures and Tables

**Table 1:** Chronological listing of clinical trials of sclerostin neutralising antibodies.

Clinical trial identifier	Phase	Study name	Number of participants and population	Treatment intervention	Primary endpoint	Study completion date (actual/estimate)
NCT01059435	I	A First-in-Human Study Evaluating AMG 785 (Romosozumab) in Healthy Men and Postmenopausal Women	74 healthy males; postmenopausal females, ages 45–59 years	Postmenopausal women: one dose of romosozumab: 0.1, 0.3, 1, 3, 5, or 10 mg/kg SC or 1 or 5 mg/kg IV or placebo SC or IV Men: one dose of romosozumab: 1 or 5 mg/kg SC or IV or placebo	Number of subjects (%) experiencing clinically significant changes in vital signs, physical exam, laboratory tests, and ECGs; developing anti-AMG 785 antibodies; reporting treatment-emergent adverse events up to 85 days after drug administration	August 2008

NCT01825785	I	A Multiple-Dose Study to Evaluate the Safety, Tolerability, Pharmacokinetics and Pharmacodynamics of AMG 785	48 healthy males and postmenopausal females (osteopenia), ages 45–80 years	1 mg/kg SC every 2 weeks × 6 doses; romosozumab or placebo 3 mg/kg every 4 weeks × 3 doses; romosozumab or placebo 2 mg/kg every 2 weeks × 6 doses; romosozumab or placebo 2 mg/kg every 4 weeks × 3 doses	Number of subjects (%) with clinically significant changes in vital signs, physical exam, laboratory tests, and ECGs; developing anti-AMG 785 antibodies; reporting treatment-emergent adverse events up to 169 days after initial drug administration	July 2009

NCT00902356	I	A First-in-Human Study Evaluating AMG 167 in Healthy Men and Postmenopausal Women	69 healthy males and postmenopausal females (osteopenia) ages 45–65 years	Postmenopausal women: one dose AMG 167 or placebo 21 mg, 70, 210, 350, or 700 mg SC or 70 mg or 350 mg IV Men: 70 mg or 350 mg SC or IV	Number of subjects (%) experiencing clinically significant changes in safety laboratory tests, physical examinations, vital signs, or ECGs; developing anti-AMG 167 antibodies; reporting treatment-emergent adverse events up to 85 days after drug administration	November 2009

NCT01742091	I	A Multiple-Dose Study of LY2541546 (Eli Lilly) in Healthy Postmenopausal Women	59 healthy postmenopausal females, ages 45–80 years	180 mg LY2541546 SC Q4W for 8 weeks or placebo at weeks 2 and 6 270 mg LY2541546 SC Q2W or Q4W for 8 weeks or placebo at weeks 2 and 6 540 mg IV Q4W for 8 weeks or placebo IV at weeks 2 and 6 750 mg LY2541546 IV Q2W for 8 weeks Placebo comparator IV or SC Q2W for 8 weeks	Number of subjects with 1+ SAE up to 141 days after drug administration	March 2010

NCT01742078	I	A Study of LY2541546 in Healthy Postmenopausal Women	60 healthy postmenopausal females, ages 45–70 years	Single dose of 7.5 mg; 25 mg; 75 mg; 225 mg; 750 mg LY2541546 IV; single dose of 150 mg LY2541546 SC; single dose of placebo administered IV or SC; single dose of 225 mg or 750 mg LY2541546 IV, OL	Number of subjects with 1+ SAE up to 85 days after drug administration	June 2010

NCT01101061	I	A Single-Dose Study Evaluating AMG 785 in Healthy Postmenopausal Japanese Women	31 Japanese postmenopausal females, ages 45–70 years	Postmenopausal Japanese women: 1 mg/kg, 3 mg/kg, or 5 mg/kg AMG 785 or placebo Postmenopausal non-Japanese women: 3 mg/kg AMG 785 or placebo	Number of subjects (%) experiencing clinically significant changes in vital signs, physical exam, laboratory tests, and ECGs; developing anti-AMG 785 antibodies; reporting treatment-emergent adverse events up to 85 days after drug administration	November 2010

NCT00950950	I	A Study to Evaluate the Effect of AMG 785 on Bone Quality of the Forearm in Postmenopausal Women With Low Bone Mass	24 healthy and postmenopausal females (osteopenia), ages 55–80 years	3 mg/kg AMG 785 or placebo SC of every 4 weeks for 3 months	Polar cross-sectional moment of inertia at the distal radius assessed by pQCT up to 169 days after drug administration	December 2010

NCT00896532	II	Phase 2 Study of AMG 785 in Postmenopausal Women With Low Bone Mineral Density	419 postmenopausal women with low BMD (*T*-score between −2.0 and −3.5), ages 55–85 years	(1) Romosozumab 70 mg, 140 mg, or 210 mg or placebo QM (2) Teriparatide 20 mcg QD (3) Romosozumab 140 mg or 210 mg or placebo every Q3M (4) Alendronate 70 mg QW (5) Denosumab 60 mg or placebo Q6M (6) Zoledronic acid 5 mg IV annually	% change from baseline at month 12 in bone mineral density at the lumbar spine for the individual AMG 785 groups and pooled placebo arms	April 2016

NCT01101048	I	Ascending-Multiple-Dose Study Evaluating AMG 167 in Healthy Men and Postmenopausal Women With Low Bone Mineral Density	74 healthy men and postmenopausal women (low BMD), ages 45–75	Postmenopausal women: AMG 167 or placebo in one of 3 fixed doses SC Healthy men: AMG 167 or placebo in one of 2 fixed doses SC (doses not specified)	Number of subjects (%) experiencing clinically significant changes in vital signs, physical exam, laboratory tests, and ECGs; developing anti-AMG 785 antibodies; reporting treatment-emergent adverse events up to 336 days after drug administration	February 2012

NCT00907296	II	A Study of AMG 785 in Tibial Diaphyseal Fractures Status Post Intramedullary Nailing	402 skeletally mature adults with a unilateral closed or Gustilo type I or II open tibial fracture and fracture fixation with intramedullary nailing, ages 18–85 years	Two, three, or four doses of AMG 785 70 mg, 140 mg, or 210 mg or four doses of placebo	Time to radiographic healing for AMG 785 versus placebo groups over 24 weeks	September 2012

NCT01406977	II	Dose Escalation Study to Evaluate the Safety and Tolerability of Multiple Infusions of BPS804 (Novartis) in Adults with Hypophosphatasia	9 healthy male and female subjects, ages 18–70 years	Dose escalation (doses not specified)	Number (%) of subjects experiencing adverse events or SAE; change from baseline in primary serological bone biomarkers up to 141 days after drug administration	September 2012

NCT01417091	II	Safety, Pharmacokinetics and Pharmacodynamics of BPS804 in Osteogenesis Imperfecta	10 male and females with osteogenesis imperfecta, ages 18–75 years	Dose escalation (doses not specified) versus placebo	Safety and tolerability; pharmacodynamic effect by means of biomarkers (days 1 and 43); change in *Z*-score from baseline to day 141	December 2012

NCT01081678	II	Study to Assess Fracture Healing with Sclerostin Antibody	332 males and females unilateral low energy intertrochanteric or femoral neck fracture as the primary injury, ages 55–95 years	Four doses of 70 mg, 140 mg, or 210 mg AMG 785 or placebo SC over 52 weeks	Functional healing as measured by the mean value for the timed-up-and-go test over weeks 6 to 20	January 2013

NCT01588509	I	Transition from Alendronate to AMG 785	60 postmenopausal women (low BMB), ages 55–85 years	Three doses of AMG 785 dose 1 or dose 2 (not specified)	Change in lumbar spine BMD from baseline at day 85	January 2013

NCT01144377	II	A Study of LY2541546 in Women with Low Bone Mineral Density	153 postmenopausal women (low BMB), ages 45–85 years	LY2541546 SC every 4 weeks with placebo given every alternate 2 weeks (patient will receive an injection every 2 weeks) for 52 weeks 180 mg or 270 mg LY2541546 SC or placebo given every 2 weeks for 52 weeks LY2541546 SC every 12 weeks or placebo every 2 weeks when LY2541546 is not administered for 52 weeks	Change from baseline to 52-week endpoint in lumbar spine BMD	February 2013

NCT01406548	II	Safety and Efficacy of Multiple-Dosing Regimens of BPS804 in Postmenopausal Women with Low Bone Mineral Density	44 postmenopausal women (low BMD), ages 45–85 years	BPS804 or placebo at dosing frequency 1, 2, or 3 (not specified)	Change from baseline in BMD in lumbar spine to month 9 in bone mineral density at the lumbar spine for the individual BPS804 groups and pooled placebo arms after 9 months; number (%) of subjects experiencing adverse events or SAE	October 2013

NCT01833754	I	Study of Romosozumab (AMG 785) Administered to Healthy Subjects and Subjects with Stage 4 Renal Impairment or Stage 5 Renal Impairment Requiring Haemodialysis	24 males and females, age ≥ 50 years (healthy, stage 4 renal impairment; end-stage renal disease)	Single-dose romosozumab SC (not specified)	Incidence of treatment-emergent adverse events; results of safety laboratory tests, vital sign measurements or ECG measurements; development of anti-romosozumab antibodies	April 2014

NCT02109042	I	A Study of Blosozumab (LY2541546) in Postmenopausal Female Participants	40 postmenopausal females >60 years	Blosozumab SC QW SC for 6 weeks	Pharmacokinetics (max concentration of blosozumab; area under the concentration curve)	July 2014

NCT02016716	III	A Randomized Phase 3 Study to Evaluate 2 Different Formulations of Romosozumab in Postmenopausal Women With Osteoporosis	294 postmenopausal women with osteoporosis and high fracture risk, ages 55–90 years	Romosozumab formulation A or B or placebo (dose, frequency not specified)	% change from baseline in DXA BMD in the lumbar spine 6 months after drug administration	December 2014

NCT01992159	II	Study with AMG 785 to Treat Japanese Women with Postmenopausal Osteoporosis	252 postmenopausal Japanese women with osteoporosis, ages 55–85 years	Romosozumab (three treatment arms, doses unknown) or placebo SC for 12 months (frequency not specified)	% changes from baseline in lumbar spine BMD at 12 months	January 2015

NCT01796301	III	An Open-Label Study to Evaluate the Effect of Treatment with AMG 785 or Teriparatide in Postmenopausal Women (STRUCTURE)	436 postmenopausal females, BMD *T*-score ≤ −2.50, ages 55–90 years	Romosozumab or teriparatide SC for 12 months	% change from baseline in DXA BMD at the total hip through month 12	January 2015

NCT02186171	III	A Double-blind Study to Compare the Safety and Efficacy of Romosozumab (AMG 785) versus Placebo in Men with Osteoporosis (BRIDGE)	245 males, BMD *T*-score ≤ 2.50 at the spine/hip, *T*-score ≤ 1.50 at spine/hip, and a history of fragility nonvertebral/vertebral fracture	Monthly SC injection of romosozumab or placebo for 12 months	% change from baseline in DXA BMD in lumbar spine at month 12	December 2016

NCT01575834	III	Registrational Study with AMG 785 to Treat Postmenopausal Osteoporosis (FRAME)	7180 osteoporotic postmenopausal females, ages 55–90 years	Romosozumab SC or placebo for 12 months, followed by SC OL denosumab for 24 months	Incidence of vertebral fracture at 12 and 24 months	February 2017

NCT01631214	III	Study to Determine the Efficacy and Safety of Romosozumab in the Treatment of Postmenopausal Women with Osteoporosis	4094 postmenopausal women with osteoporosis at high risk for fracture: hip BMD *T*-score ≤ −2.5 SD and a vertebral fracture or hip BMD *T*-score ≤ −2.0 SD and a recent hip fracture or two vertebral fractures, ages 55–90 years	Romosozumab SC injections and placebo alendronate (oral) for 12 months followed by OL alendronate (oral) for at least another 12 months (until end of study) Oral alendronate and placebo AMG 785 subcutaneous injections for 12 months, followed by open-label alendronate (oral) for at least another 12 months (until end of study)	Incidence of clinical fracture or new vertebral fracture at 24 months after drug administration	May 2017

BMD: bone mineral density; ECG: electrocardiogram; IV: intravenously; OL: open label; SAE: serious adverse events; SC: subcutaneously; QW: weekly; Q2W: once every 2 weeks; Q4W: once every four weeks; QD: daily; QM: every month.
